# SARS-CoV-2 antibody responses associate with sex, age and disease severity in previously uninfected people admitted to hospital with COVID-19: An ISARIC4C prospective study

**DOI:** 10.3389/fimmu.2023.1146702

**Published:** 2023-03-15

**Authors:** Eleanor Parker, Jordan Thomas, Kelly J. Roper, Samreen Ijaz, Tansy Edwards, Federica Marchesin, Ksenia Katsanovskaja, Lauren Lett, Christopher Jones, Hayley E. Hardwick, Chris Davis, Elen Vink, Sarah E. McDonald, Shona C. Moore, Steve Dicks, Keerthana Jegatheesan, Nicola J. Cook, Joshua Hope, Peter Cherepanov, Myra O. McClure, J. Kenneth Baillie, Peter J. M. Openshaw, Lance Turtle, Antonia Ho, Malcolm G. Semple, William A. Paxton, Richard S. Tedder, Georgios Pollakis, ISARIC4C Investigators

**Affiliations:** ^1^ Department of Infectious Disease, Imperial College London, London, United Kingdom; ^2^ National Institute of Health and Care Research (NIHR) Health Protection Research Unit in Emerging and Zoonotic Infections, Department of Clinical Infection, Microbiology and Immunology, Institute of Infection, Veterinary and Ecological Sciences, University of Liverpool, Liverpool, United Kingdom; ^3^ Blood Borne Virus Unit, Reference Department, UK Health Security Agency, London, United Kingdom; ^4^ Medical Research Council (MRC) International Statistics and Epidemiology Group, London School of Hygiene and Tropical Medicine, London, United Kingdom; ^5^ Medical Research Council, University of Glasgow Centre for Virus Research, Glasgow, United Kingdom; ^6^ National Health Service (NHS) Blood and Transplant, London, United Kingdom; ^7^ Chromatin Structure and Mobile DNA Laboratory, The Francis Crick Institute, London, United Kingdom; ^8^ Roslin Institute, University of Edinburgh, Edinburgh, United Kingdom; ^9^ National Heart & Lung Institute, Imperial College, London, United Kingdom

**Keywords:** SARS-CoV-2, immunology, COVID-19, virus, disease, serology, neutralisation

## Abstract

The SARS-CoV-2 pandemic enables the analysis of immune responses induced against a novel coronavirus infecting immunologically naïve individuals. This provides an opportunity for analysis of immune responses and associations with age, sex and disease severity. Here we measured an array of solid-phase binding antibody and viral neutralising Ab (nAb) responses in participants (n=337) of the ISARIC4C cohort and characterised their correlation with peak disease severity during acute infection and early convalescence. Overall, the responses in a Double Antigen Binding Assay (DABA) for antibody to the receptor binding domain (anti-RBD) correlated well with IgM as well as IgG responses against viral spike, S1 and nucleocapsid protein (NP) antigens. DABA reactivity also correlated with nAb. As we and others reported previously, there is greater risk of severe disease and death in older men, whilst the sex ratio was found to be equal within each severity grouping in younger people. In older males with severe disease (mean age 68 years), peak antibody levels were found to be delayed by one to two weeks compared with women, and nAb responses were delayed further. Additionally, we demonstrated that solid-phase binding antibody responses reached higher levels in males as measured *via* DABA and IgM binding against Spike, NP and S1 antigens. In contrast, this was not observed for nAb responses. When measuring SARS-CoV-2 RNA transcripts (as a surrogate for viral shedding) in nasal swabs at recruitment, we saw no significant differences by sex or disease severity status. However, we have shown higher antibody levels associated with low nasal viral RNA indicating a role of antibody responses in controlling viral replication and shedding in the upper airway. In this study, we have shown discernible differences in the humoral immune responses between males and females and these differences associate with age as well as with resultant disease severity.

## 1 Introduction

Individual risk of COVID-19 severity is heterogenous and determined by several factors including the host’s clinical characteristics and genetics (1–4). The most important predictors of severe disease are advanced age and male sex followed by the presence of co-morbidities including cardiac disease, metabolic disorders such as obesity and diabetes, hypertension and respiratory diseases (2, 5–11). Further, recent studies have identified several genetic correlates of disease severity (4, 12–14).

Disease outcome may also be determined by the timing and magnitude of humoral immune responses (15–19). Generally, antibody responses to acute infection in SARS-CoV-2-naïve individuals are rapid; the majority of patients seroconvert for virus-specific IgM and then IgG between 10-19 days post-symptom onset (20–22). The primary viral targets of humoral responses to SARS-CoV-2 are the Spike (S) glycoprotein (including the RBD domain) and the nucleocapsid (N) protein (23). The majority of virus neutralisation activity is provided by antibodies directed against the receptor binding domain (RBD) of the spike protein S1 sub-unit, which blocks the interaction between S and ACE2 (24–27). Mild cases of COVID-19 have previously been associated with higher ratios of antibodies directed against RBD as opposed to N, as well as rapid reduction of respiratory tract viral RNA concomitant with rises in anti-RBD IgG (16, 24). Faster production of both total and RBD-specific IgG has been observed in female patients (28, 29), and early upregulation of specific IgM responses (24, 30) and neutralising RBD specific responses (31) have been associated with improved disease outcome. In response to vaccination, elderly patients generate weaker humoral responses, characterised by slower induction of antibody production, lower magnitude Ab titres at peak and quicker Ab decline, when compared to younger adults (32–35). Whilst several reports have shown that elderly patients are able to generate robust and neutralising antibody responses during acute infection (7, 36, 37), there is less evidence of early antibody kinetics impacting on disease outcome in elderly patients.

Using serum samples from patients hospitalised during the first wave of the COVID-19 pandemic in the United Kingdom (UK), we have performed an extensive analysis of the serological responses generated to SARS-CoV-2 in an immune-naïve population. Anti-RBD reactivity, neutralising function and class specific antibodies to S and N proteins were measured using a hybrid double antigen binding assay (DABA) (38), a pseudo-virus particle (PVP) neutralisation assay and Ig capture assays respectively. This portfolio of assay formats was used previously in the characterisation of the antibody response kinetics in Ebola virus survivors following the Sierra Leone outbreak of 2014-2016 (38, 39). By comparing serological responses in hospitalised patients of different age groups and sexes in the context of the early UK outbreak when the virus population was relatively homogenous, we have been able to identify host characteristics that contribute to the risk of severe disease. Additionally, repeat sampling starting from early in hospital admission through to convalescence has provided greater insights into the influence of sex and age on early antibody kinetics, and their association with outcome.

## 2 Materials and methods

### 2.1 Study cohort patients and samples

This analysis included sera from 337 patients admitted to UK hospitals with COVID-19 between February and June 2020 before vaccines were made available and therefore describing a new infection in a naïve human population. The patients were enrolled in the International Severe Acute Respiratory and emerging Infections Consortium (ISARIC) World Health Organization (WHO) Clinical Characterisation Protocol UK (CCP-UK) study. Study participants were confirmed SARS-CoV-2 positive by reverse transcription polymerase chain (PCR) reaction or were highly suspected cases based on clinical presentation and providing a serological response in one or more of the described assays being recorded. Acute infection samples were collected within 21 days of the onset of symptoms and convalescent samples were collected when SARS-CoV-2 PCR showed undetectable viral burden. A number of patients underwent serial sampling (2/n=129, 3/n=91, 4/n=12, 5/n=1), with not all follow up specimens tested in every assay implemented. Samples with repeated measures were included in a mixed effect regression model to analyse the antibody responses over time (section 3.5).

Patients were stratified into five categories of peak illness severity based on the World Health Organization (WHO) COVID-19 ordinal scale (40): 1) no oxygen requirement (WHO score 3); 2) patient requiring oxygen by face mask or nasal prongs (WHO score 4); 3) patient requiring high-flow nasal oxygen (HFNO) or non-invasive ventilation (NIV) (WHO score 5); 4) patients requiring mechanical ventilation (WHO score 6/7) and 5) patients who died within 28 days. (WHO score 8).

### 2.2 Anti-SARS-CoV-2 S1, spike and NP IgM and IgG capture ELISAs

Three viral antigens all based on the hCoV-19/Australia/VIC01/202 (Accession MT007544) lineage were tested. The SARS-CoV-2 full length spike glycoprotein (Spike/amino acids 1–1211; His-tag) and the nucleoprotein (NP) conjugated to Horseradish peroxidase (HRP) were purchased from The Native Antigen Company (Kidlington, Oxford, UK). The SARS-CoV-2 S1 antigen (spanning Wuhan-Hu-1 SARS-CoV-2 Spike residues 1–530, C-terminal twin Strep tag) (41, 42) was produced and gifted by The Francis Crick Institute and conjugated to HRP using the Bio-Rad LYNX HRP conjugation kit, in accordance with the manufacturer’s instructions. Recombinant NP antigens from seasonal coronavirus NL63, OC43, HKU1 and 229E were used to block non-specific NP responses as previously described (43). These proteins were produced in *Escherichia coli* with N-terminal hexahistidine-SUMO and C-terminal Twin Strep tags and purified by tandem immobilised metal and StrepTactin^®^ affinity chromatography. The IgM and IgG capture ELISAs for the detection of antibody to S1, Spike and NP were undertaken as described previously (43).

### 2.3 SARS-CoV-2 RNA quantitative reverse transcriptase polymerase chain reaction

SARS-CoV-2 RNA was quantified using a NEB Luna Universal Probe One-Step RT-qPCR Kit (New England Biolabs, E3006) and 2019-nCoV CDC N1 primers and probes (IDT, 10006713)). Genome copy numbers were quantified using a standard curve generated from serial dilutions of a plasmid containing the target N protein gene fragment. The standard was quantified and quality controlled using QX600 droplet digital PCR system (Bio-rad, UK).

### 2.4 Anti-RBD hybrid DABA immunoassay

Antibodies targeting SARS-CoV-2 were measured using a hybrid double antigen bridging assay (DABA) that was previously developed to detect Ebola virus (EBOV) glycoprotein targeting antibodies (38) and recently adapted and validated to detect SARS-CoV-2 directed antibodies, using the same methodology for performance and analysis as described previously (44). Briefly, an S1 antigen coated onto a solid phase was used to bind all reactive immunoglobulins present in a sample, after a which an HRP conjugated RBD antigen was added to detect antibody binding which was expressed as arbitrary units (AU)/ml (44). Owing to the use of an antigen as the detector, the DABA detects all classes of antibody that target a specific antigen, unlike methods which discriminate between IgM or IgG.

### 2.5 Generation of SARS-CoV-2 pseudovirus particle, infectivity and neutralisation assay

#### 2.5.1 Cell culture

HEK293T (ATCC^®^ CRL-3216^™^) cells were cultivated in Dulbecco’s modified eagle medium (Invitrogen) and supplemented with 10% heat-treated FCS (Sigma), 2mM/ml L-glutamine (Invitrogen), 100 U/ml penicillin (Invitrogen) and 100 mg/ml streptomycin (Invitrogen), termed complete DMEM (Thermofisher). HEK293T/ACE-2 cells were used to monitor PVP infectivity and in performing serum neutralisation assays. All cells were cultured at 37°C and at 5% CO_2._

#### 2.5.2 SARS-CoV-2 PVP production and infection

The ancestral SARS-CoV-2 S glycoprotein (Accession MN908947) was cloned into the pCDNA3.1 expression plasmid (produced by GeneArt Gene Synthesis) and was used in generating PVP stocks *via* a lentiviral system to generate single-cycle infectious viral particles as previously described (45, 46). HEK293T cells (5.0x10^5^ in each well of a 6-well tissue culture flask) (Corning) were grown in 2.0 ml of complete DMEM overnight. Cells were transfected with 750 ng of the lentiviral luciferase reporter construct, pCSFLW, along with 450 ng of the SARS-CoV-2 S expression plasmid and 500 ng of the lentiviral backbone, p8.91, using cationic polymer transfection reagent (Polyethylenimine) (Polysciences) and in the presence of OptiMEM (Invitrogen). OptiMEM/plasmid mix was removed 16 h post transfection and 2.0 ml complete DMEM added with the single-cycle infectious SARS-CoV-2 stock harvested 48 h later, passed through a 0.45µM filter, aliquoted and stored at −80°C. PVP infection was monitored on HEK293T/ACE-2 cells through measuring luciferase activity (expressed from the HIV-1 LTR promoter) under control of Tat expression from the HIV-1 backbone. 100 µl of virus stock was used to infect 1.5x10^4^ cells/well for 6 h in a white 96 well plate (Corning). Following infection 100 µl DMEM complete medium was added to each well. 48 h post infection, media was discarded from the wells and the cells washed with PBS (Thermofisher), lysed with 30 µl cell lysis buffer (Promega) and luciferase activity determined utilising the commercially available luciferase assay (Promega) and measured using a BMGLabtech FluoroStar Omega luminometer.

#### 2.5.3 SARS-CoV-2 S PVP neutralisation assay

SARS-CoV-2 enveloped PVP was thawed and pooled and subsequently diluted 1/20 in complete DMEM. Serum samples from SARS-CoV-2 individuals were serially diluted 2-fold with complete DMEM; 28 µl serum dilution was incubated with 420 µl diluted SARS-CoV-2 PVP for 30 min at RT. 200 µl of virus/serum dilution mix was used to infect HEK293T/ACE-2 cells. Luciferase activity readings of neutralised virus were analysed i) by considering 0% inhibition as the infection values of the virus in the absence of convalescent plasma included in each experiment, ii) by considering 0% inhibition as the infection values of two consecutive high dilutions not inhibiting virus entry. The neutralisation activity defined as the serum dilution that reduced viral infectivity by 50%, 70% or 90% (IC_50_, IC_70_ or IC_90_, respectively).

### 2.6 Statistical analyses

Statistical analyses were performed using GraphPad Prism 6.0 software. Unpaired sample comparisons were conducted for all data; however, individual figures state the corresponding statistical test performed. These include parametric and non-parametric t-tests (student t-test and Mann-Whitney U test) and non-parametric ANOVA (Kruskal-Wallis test). Significant P values < 0.05 were depicted by * or a horizontal line above the groups compared. Repeated measures linear regression was used to model antibody levels over time, including a random intercept term to account for within-individual correlation, age and a time-sex interaction to predict trajectories for males and females separately, adjusted for age.

## 3 Results

### 3.1 Patient demographics

**Figure 1:**
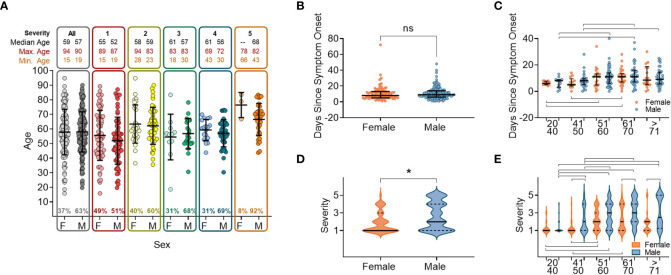
Sex and age distribution within groups with relation to days since disease onset and severity. **(A)** Number of individuals, female (F) or male (M), overall and when broken down into disease severity groupings (S1-S5). **(B)** Days since symptom onset split into females (orange) and males (orange) for all individuals. **(C)** Days since symptom onset split into females and males and relative to age groupings. **(D)** Disease severity split into females and males for all individuals. **(E)** Disease severity split into females and males relative to age groupings. In all panels mean values and confidence intervals shown (black lines). Lines above or below the groups indicate significant differences between groups as found by implementing a paired t-test or a non-parametric ANOVA (Kruskal-Wallis test). * indicates statistical significance P < 0.05.

We analysed the patient demograhics of individuals within our cohort, specifically age and sex, to determine the risk of severe disease across these groups. A higher proportion of the 337 study participants were male (63.0%, n=210). Median age was 57 years (range: 15–94) with no age difference observed between sexes (male median age = 57.3 years/range: 19–90 and female median age = 57.7 years/range: 15–94). As this was a hospital study, no asymptomatic individuals were enrolled. Participants were grouped into categories S1-S5 according to disease severity (**Supplementary Figure 1**) (40). The ratio of males to females increased within the higher disease severity groupings, from 47% of participants in S1, to 66% of participants in S4, and with only three females (8.1%) in S5 (individuals that died within 28 days of disease onset) (**Figure 1A**). There were no age differences between sexes within severity groupings, and the age range narrowed as disease severity increased (**Figure 1A**). The average participant age across severity groups was similar with S5 being an exception, where participants tended to be older.

We next anlysed the time between the onset of symptoms and hospital presentation to compare the rate of deteriation across different patient groupings. No difference was found between males and females in the time between symptom onset and hospital presentation (**Figure 1B**). When the cohort was stratified by 10 yearly age categories, participants between 50 and 70 years old were recruited later than participants <50 years or >70 years (**Figure 1C**), reflecting a delay from disease onset to when participants presented at the hospital. In this cohort we found that overall, males developed more severe disease than females (**Figure 1D**), which was shown in all age categories above 50 years (**Figure 1E**).

### 3.2 Antibody responses by gender and age

**Figure 2:**
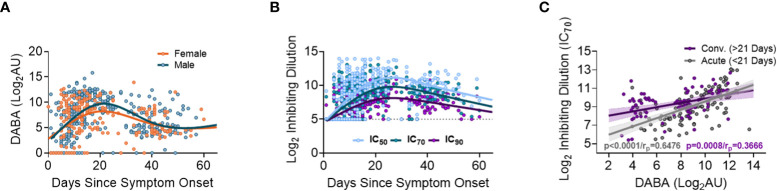
Association between anti-RBD as well Ab neutralisation responses with days since disease onset. **(A)** Anti-RBD binding in relation to days since disease onset and split into females (orange) and males (blue). The lines (females orange and males blue) show the spline/LOWESS curves indicating the overtime evolutionary trend of the data. **(B)** Neutralisation antibody responses depict in relation to days since disease onset and curves representing the spline/LOWESS for the IC_50_, IC_70_ and IC_90_ values indicating the overtime evolution trend. **(C)** Association between anti-RBD and neutralisation responses (IC_70_). Spearman correlation test (P<0.0001/r_p_=0.6476), in acute infection (under 21 days) and (P<0.0001/r_p_=0.3666) in convalescence (over 21 days).

When measuring anti-RBD using the hybrid DABA (an antibody class neutral assay) high antibody levels were measured within one week following onset of symptoms and were maintained at high levels for 3 to 4 weeks (**Figure 2A**). Anti-RBD titres reached a peak around day 21 following symptom onset for both males and females, and peak antibody levels were higher in males. Neutralising antibodies (nAb) (IC_50_, IC_70_ or IC_90_), measured using the PVP neutralisation assay, revealed a similar serological profile to anti-RBD with a sharp initial increase reaching the peak at around day 26 post symptom onset (**Figure 2B**). When comparing anti-RBD with nAb responses (IC_70_) a correlation was observed during the first 21-day period (P<0.0001, r_p_=0.6476). This correlation remained but was lower in magnitude after 21 days following disease onset (P<0.0001, r_p_=0.3666) (**Figure 2C**).

At recruitment to the study, corresponding to the time that a participant was hospitalized, no significant differences were identified between males and females in anti-RBD (DABA) or nAb responses (**Supplementary Figures 2A, B**). However, when divided into age groups, significant differences were observed in the antibody responses between age groupings for both males and females (**Supplementary Figures 2C, D**). Specifically, individuals between 51-70 years of age demonstrated higher anti-RBD levels and nAb responses (IC_70_) than those aged 20-49 or those >70 years old. (**Supplementary Figures 2C, D**).

We further studied responses against the two main immunogenic viral proteins, the spike and the non-envelope nucleoprotein (NP). The S1 region of spike that includes the RBD was was also studied individually considering it is the primary target of nuetralising antibodies. In samples taken at recruitment, which represents a range of days between patients since the onset of symptoms and hospital presentation, IgM and IgG antibody binding responses to spike, S1 and NP were not significantly different between males and females for most age groupings, except for the IgM responses to S1, which were higher in men aged 60-70 (**Supplementary Figures 3A-F**). The IgM responses to the S1, Spike, and NP proteins all demonstrated higher levels in individuals aged between 41-60 in comparison to the <40 or >70 age groupings (**Supplementary Figures 3A-C**, respectively), with a similar profile observed for IgG (**Supplementary Figures 3D-F**, respectively).

Overall, when comparing antibody responses (DABA, neutralizing, IgG and IgM) at recruitment no differences were found between males and females within age categories but differences were observed between the different age categories. Individuals in age categories 20-40 and >70 had lower antibody titres than those in the intermediate age categories.

### 3.3 Total, neutralizing and class Ab associations

We next analysed the relationship between the antibody classes IgM and IgG against different virus antigens, comparing acute infection with convalescence. During acute infection, IgG responses against Spike protein correlated with IgM antibody levels (P<0.0001), whereas this correlation disappeared during convalescence (**Supplementary Figure 4A**). This association was not observed when comparing IgG versus IgM responses against S1 or NP antigens during acute infection or convalescence (**Supplementary Figures 4B, C**, respectively), indicating that antibody class induction is variable across different antigens. Strong correlations were found between Spike-IgM and S1-IgM as well as between Spike-IgG and S1-IgG responses (**Supplementary Figures 4D, E**) with again no difference between acute infection and convalescence. In contrast, weak correlations were observed when comparing NP with Spike or S1 antibody responses (**Supplementary Figures 4F-I**).

There were signficant correlations between total anti-RBD binding (DABA) and both IgM and IgG to total spike and S1 (**Supplementary Figures 5A-D**) during the acute infection phase (<21 days post-symptom onset), which became weaker or not significant during convalescence (>21 days post-symptom onset) for IgG, but not IgM. A similar pattern was observed for the correlation between anti-RBD binding and anti-NP binding, indicating that the anti-RBD binding correlated to some extent with the total antibody response, though the magnitude was less for binding to NP (**Supplementary Figures 5E, F**).

Next, we compared antibody classes IgG and IgM against Spike, NP and S1 to nAb responses (IC_70_) directed against the same antigens. We observed similar profiles during both acute infection and convalescence (**Supplementary Figures 5G-L**). Collectively, these results suggest that total antibody, as well as class-specific responses (all measured by solid-phase binding ELISA), correlate with nAb activity induced in early infection. The most notable associations between responses were observed when comparing Spike, S1 IgG or IgM levels with nAb responses (**Supplementary Figures 5G-J**). This would indicate that both IgM and IgG induced during acute infection and convalescence are associated with virus neutralisation with spike, including the RBD domain as the predominant target.

### 3.4 Antibody levels and neutralisation associate with disease severity over time

**Figure 3:**
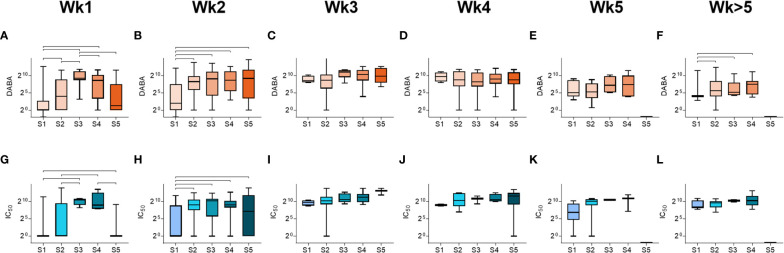
Antibody responses by severity groupings and time following disease onset. **(A-F)** Total anti-RBD titres grouped by severity (S1-S5), measured from samples taken at **(A)** week 1, **(B)** week 2, **(C)** week 3, **(D)** week 4, **(E)** week 5 and **(F)** past week 5 post-symptom onset. **(G-L)** Neutralising antibody (IC_50_) titres grouped by severity (S1-S5), measured from samples taken at **(G)** week 1, **(H)** week 2, **(I)** week 3, **(J)** week 4, **(K)** week 5 and **(L)** past week 5 post-symptom onset.Statistically significant differences (non-parametric ANOVA (Kruskal-Wallis test) are indicated by horizontal lines above the groupings.

We next analysed the relationships between anti-RBD and nAb responses (IC_70_) with disease severity (**Figure 3**). In all severity groups, antibody levels increased over time, but initially relatively lower levels were observed in groups S1 and S5 in week 1, particularly for nAb responses, when compared to intermediate severity groups (**Figure 3** and **Supplementary Figure 6**). By week 3, high levels of anti-RBD and nAbs were measured in all groups, and maintained for the duration of the study period (**Figure 3**). A similar profile was observed when comparing IgM and IgG responses for Spike, NP and S1 (**Supplementary Figure 7**). These results indicate that whilst antibody levels rise with time in all severity groups, individuals in the most severe and least severe disease groups developed antibody responses more slowly than those in intermediate groupings.

### 3.5 Differing profiles of antibody responses over time in male and female pariticipants

**Figure 4:**
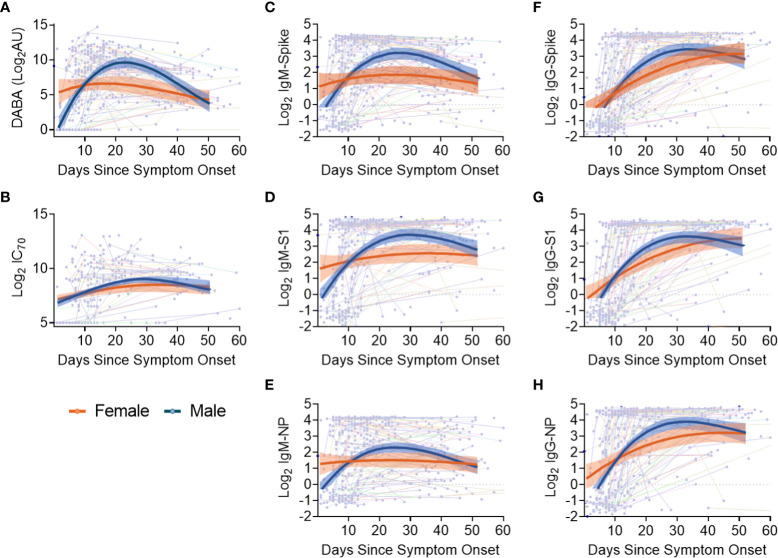
Evolution in time of antibody titres following disease onset by sex and subclass. **(A)** Anti-RBD titres. **(B)** nAb (IC_70_) responses. **(C-E)** IgM binding responses against spike **(C)**, S1 **(D)** and NP **(E)**. **(F-H)** IgG binding responses against spike glycoprotein **(F)**, S1 **(G)** and NP **(H)** responses. The thin lines in background indicate individuals with longitudinal samplings with each dot representing a time point collection. For all panels, best-fit curves with 95% confidence intervals are shown for females (orange) and males (blue).

Sex differences in antibody responses over time were investigated using a mixed effect regression model comparing different antibody measurements. Female participants demonstrated higher initial anti-RBD responses which declined slowly from day 20, whilst male participants had lower early anti-RBD responses that sharply increased up until day 30 before falling to similar levels as females at 50 days post symptom onset (**Figure 4A**). However, when comparing nAb (IC_70_) responses over the same period (**Figure 4B**), similar antibody profiles were found for both males and females, suggesting that the higher anti-RBD responses measured by the hybrid DABA observed in males were not associated with higher neutralisation. When comparing IgM and IgG Ab responses against Spike, S1 or NP antigens over the 50 days period following symptom onset, a very similar profile was observed to DABA anti-RBD measurements (**Figures 4C-H**). However, the most marked differences were observed with IgM between males and females (**Figures 4C-E**) and especially for the Spike and S1 protein (**Figures 4C, D**, respectively). These results highlight the differences in antibody response kinetics between male and female participants and in particular in early IgM responses targeted to the dominant antigens for neutralisation.

### 3.6 Upper respiratory tract SARS-CoV-2 viral RNA in relation to demographics, disease severity and Ab responses

**Figure 5:**
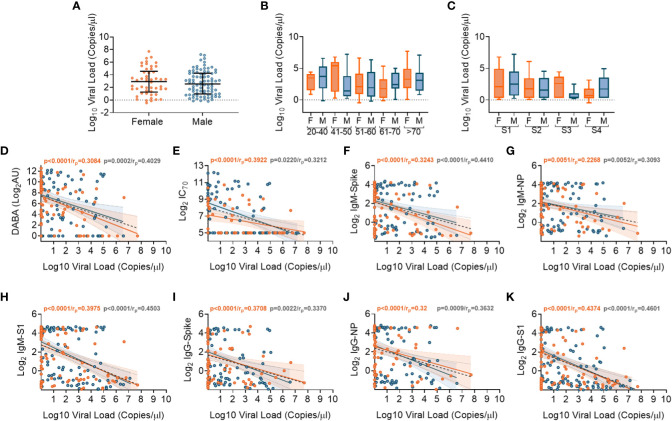
Association of SARS-CoV-2 upper respiratory tract viral loads in relation to sex, age and Ab responses at time of sampling. **(A)** Overall viral load measurements in relation to sex. **(B)** Viral loads according to age groupings and between females (orange) and males (blue). **(C)** Viral loads according to disease severity groupings (S1-S4) and between females (orange) and males (blue). **(D)** Associations between viral loads and overall DABA anti-RBD binding responses. **(E)** Associations between viral loads and neutralisation antibody (IC_70_) responses. **(F-H)** Associations between viral loads and IgM antibody binding responses against spike **(F)**, NP **(G)** and S1 **(H)** antigens. **(I-K)** associations between viral loads and IgG antibody binding responses against spike **(I)**, NP **(J)** and S1 **(K)** antigens. **(D-K)** Inverse correlations shown (black dotted line) with males shown in blue and females in orange.

We performed SARS-CoV-2 viral transcript measurements on upper respiratory tract samples, taken from 174 participants, at a median of 14 days from date of symptom onset (IQR8-30). There were no differences in viral RNA levels by sex (**Figure 5A**), nor by age or disease severity (**Figures 5B, C**, respectively). Viral RNA copy number fell over time from symptom onset (**Supplementary Figures 8A, B**), but the number of days from symptom onset to when participants first presented at hospital and were sampled at study recruitment did not vary according to age or disease severity (**Supplementary Figures 8C, D**). We next aimed to identify whether there were associations between viral RNA load and the array of antibody responses previously described. Contemporaneously collected samples showed an inverse correlation between viral RNA measurements and anti-RBD and nAb titres (IC_70_), (**Figures 5D, E**, respectively). Similar inverse correlations were observed when comparing Spike, NP and S1 antigen directed IgM (**Figures 5F-H**, respectively) and IgG (**Figures 5I-K**, respectively). The results indicate that the presence of antibody responses were associated with a reduction in nasal levels of viral RNA, with no difference by sex.

## 4 Discussion

This study of individuals during the early stages of the pandemic (February-May 2020), using several measurements of host responses and viral RNA, has enabled the identification of differences in antibody profiles in an immunologically naïve population. Very early in the SARS-CoV-2 pandemic it was reported that a number of factors such as age, sex, co-morbidities, obesity and ethnicity were associated with the risk of severe disease (2, 5–7). In our cohort, analysis of patient demographics and disease severity showed that males were disproportionately represented in higher severity groups, especially in the age groupings above 50. Further, we showed that 90% of participants who died (severity group 5) were male with a median age of 68, supporting previous reports in which older males were more prone to death (47). Nevertheless, we observed no differences in the mean age between males and females when grouped by disease severity, potentially indicating that age is a stronger determinant of disease severity than sex.

Many other studies have measured antibody responses following acute infection with SARS-CoV-2 (23, 24, 31, 48–50). However, most were either cross-sectional, did not measure such early responses or do not utilise a multitude of comparable antibody assays. Therefore, a strength of this study was the use of an array of assays to measure antibody responses against the two main immunogenic viral proteins S and NP (48). Three different types of binding assays were performed with one quantifying total antibodies against RBD (DABA) and the two other measuring IgM and IgG responses against Spike, S1 and NP. Additionally, a PVP neutralisation assay was also employed to assess the functionality of the antibodies generated. Through comparing these different measurements, we observed an overall robust correlation between binding antibody titres (measured by DABA or ELISA), regardless of IgM or IgG class, to neutralising antibodies which is not affected by age, gender or disease severity. Comparison of total anti-RBD antibodies, as measured by DABA, with IgG and IgM Spike and S1 directed antibodies highlighted a strong correlation between these measurements during the acute infection phase (**Supplementary Figures 5A-D**). However, this correlation became significantly weaker when comparing anti-RBD antibodies to spike and S1 directed IgG antibodies during the convalescent phase (**Supplementary Figures 5A-D**), indicating a strong contribution of IgM to the antibody responses measured by DABA and suggesting a progressive switch to IgG as the predominant class of spike directed antibodies. Similarly, we observed a strong correlation between Spike and S1 directed IgM and IgG antibody responses with nAbs during both acute infection and convalescence, suggesting that both early IgG and IgM posess neutralising activity (**Supplementary Figures 5G-J**), as has been previously reported (51–53). Together, these results further highlight how this multi-faceted analysis can reveal the evolvoing dynamics serological responses within patients. The associations between different antibody classes and functions observed in this study can be used to provide retrospective insights into humoral immunity in the most vulnerable population during the early stages of the pandemic. Such associations can facilitate further understanding of how inital immune responses can evolve over a pandemic of a novel virus, when population immune responses are not primed by previous exposures or vaccination.

We sought to identify how the timing of antibody responses associates with disease severity. Our data supports previous findings that antibody seroconversion occurs 10-19 days post symptom onset (20–22, 30, 49, 54) and with higher IgM than IgG antibody titres measured during acute infection (**Supplementary Figures 4A-C**). Despite some differences in the rate of induction of antibody response between males and females (discussed below), we showed that total anti-RBD as well as nAb responses peaked around 3 weeks post-symptom onset for both sexes and across all age groupings. Through comparing antibody titres at hospital presentation in different age groups, we showed that there were higher levels of IgM targeting spike, S1 and NP in indivudals aged between 41-60 than in other age groups (**Supplementary Figure 3**). Similarly, we also showed that both anti-RBD and nAb responses to all antigens tested were delayed in individuals with lowest disease severity, as previously reported (50, 54, 55) and in those with the highest severity (fatal outcome) (**Figures 3**, **4** and **Supplementary Figure 6**, respectively). However, patients in the 51 to 60 age group were recruited up to 4 days later in disease onset than the other groups (**Figure 1C**), which may account for some of the differences observed. Nevertheless, these data, together with the finding that older males are more prone to severe disease and death, suggests that delayed antibody production is associated with severe disease and death in older patients (>60) but not in younger individuals (<40). A potential explanation for this disparity is that in younger individuals, more robust innate immune responses help to limit virus replication during early infection, reducing the overall viral burden and subsequently delaying the production of Ab responses. Conversely, advanced age is associated with blunted innate immune responses, which in combination with delayed Ab production likely accounts for the higher risk of severe disease. Indeed, delayed and impaired type 1 IFN responses have been associated with risk of severe COVID-19 (56) and these responses are known to be dysregulated in elderly individuals, contributing to the age related discrepancies in patient outcome (57–59).

When comparing antibody responses between sexes, we observed a more rapid induction of antibody responses in females than was observed in male participants and have associated this with differences in disease severity. Therefore, it is possible that a contributing factor to sex-associated differences in disease severity is the timing of antibody responses, whereby a delay in antibody production may account for increased risk of severe disease outcome. This association between age, sex and disease outcome with antibody kinetics has been previously reported, where females demonstrated more rapid increases in protective IgG responses than males (29) and that in severe cases, females had higher concentrations of virus-specific IgG (28). Here, we identify that the timing of measuring serological responses is important when correlating to disease status and outcome. This should be taken into consideration when comparing results to other studies where levels of IgM have reported contradictory findings between the sexes (60).

Through measuring upper respiratory tract viral RNA transcripts, indicative of localised viral shedding and therefore a surrogate measure for viral load, we observed an inverse correlation between nAb levels which may indicate a critical role of effective serological responses limiting viral replication and leading to clearance of the infection. Nevertheless, our samples were obtained a median of 2 weeks post symptom onset and therefore viral RNA has been predominantly measured during the decline phase of infection (61). Additionally, it is possible that this observation could be a non-causal association with emergence of effective cellular immunity. It should also be noted that viral load in the lower respiratory tract, which may play an important role in defining disease severity, was not measured in this study. Additionally, a formal analysis of the avidity of the anti-RBD serological response following recovery has not been undertaken. Preliminary unpublished data indicate avidity is low after recovery from infection but greatly increased after vaccine administration.

In this study, immunological linkages with disease outcome have been deciphered independently in a naïve host population and with a homogenous viral strain. The analyses of patients early in the pandemic has been vital in enabling description of the associations we have identified. Subsequent multiple exposures to different types of vaccines, natural infections and the emergence of diverse viral variants makes unravelling further host genetic and immune factors associated with disease challenging, meaning that the data presented here are unique, and are unlikely to be obtained as the pandemic evolves.

## Data availability statement

The raw data supporting the conclusions of this article will be made available by the authors, without undue reservation.

## Ethics statement

This analysis included sera from 337 patients admitted to UK hospitals with COVID-19 between February and June 2020 and enrolled in the International Severe Acute Respiratory and emerging Infections Consortium (ISARIC) World Health Organization (WHO) Clinical Characterisation Protocol UK (CCP-UK) study. The patients/participants provided their written informed consent to participate in this study.

## ISARIC4C Investigators

Consortium Lead Investigator: J Kenneth Baillie; Chief Investigator: Malcolm G Semple; Co-Lead Investigator: Peter JM Openshaw; ISARIC Clinical Coordinator: Gail Carson *Co-Investigator*: Beatrice Alex, Petros Andrikopoulos, Benjamin Bach, Wendy S Barclay, Debby Bogaert, Meera Chand, Kanta Chechi, Graham S Cooke, Ana da Silva Filipe, Thushan de Silva, Annemarie B Docherty, Gonçalo dos Santos Correia, Marc-Emmanuel Dumas, Jake Dunning, Tom Fletcher, Christoper A Green, William Greenhalf, Julian L Griffin, Rishi K Gupta, Ewen M Harrison, Julian A Hiscox, Antonia Ying Wai Ho, Peter W Horby, Samreen Ijaz, Saye Khoo, Paul Klenerman, Andrew Law, Matthew R Lewis, Sonia Liggi, Wei Shen Lim, Lynn Maslen, Alexander J Mentzer, Laura Merson, Alison M Meynert, Shona C Moore, Mahdad Noursadeghi, Michael Olanipekun, Anthonia Osagie, Massimo Palmarini, Carlo Palmieri, William A Paxton, Georgios Pollakis, Nicholas Price, Andrew Rambaut, David L Robertson, Clark D Russell, Vanessa Sancho-Shimizu, Caroline J Sands, Janet T Scott, Louise Sigfrid, Tom Solomon, Shiranee Sriskandan, David Stuart, Charlotte Summers, Olivia V Swann, Zoltan Takats, Panteleimon Takis, Richard S Tedder, AA Roger Thompson, Emma C Thomson, Ryan S Thwaites, Lance CW Turtle, Maria Zambon; *Project Manager*: Hayley Hardwick, Chloe Donohue, Fiona Griffiths, Wilna Oosthuyzen; *Project Administrator*: Cara Donegan, Rebecca G. Spencer; *Data Analyst*: Lisa Norman, Riinu Pius, Thomas M Drake, Cameron J Fairfield, Stephen R Knight, Kenneth A Mclean, Derek Murphy, Catherine A Shaw; *Data and Information System Manager*: Jo Dalton, Michelle Girvan, Egle Saviciute, Stephanie Roberts, Janet Harrison, Laura Marsh, Marie Connor, Sophie Halpin, Clare Jackson, Carrol Gamble, Daniel Plotkin, James Lee; *Data Integration and Presentation*: Gary Leeming, Andrew Law, Murray Wham, Sara Clohisey, Ross Hendry, James Scott-Brown; *Material Management*: Victoria Shaw, Sarah E McDonald. *Patient Engagement*: Seán Keating; *Outbreak Laboratory Staff and Volunteers*: Katie A. Ahmed, Jane A Armstrong, Milton Ashworth, Innocent G Asiimwe, Siddharth Bakshi, Samantha L Barlow, Laura Booth, Benjamin Brennan, Katie Bullock, Benjamin WA Catterall, Jordan J Clark, Emily A Clarke, Sarah Cole, Louise Cooper, Helen Cox, Christopher Davis, Oslem Dincarslan, Chris Dunn, Philip Dyer, Angela Elliott, Anthony Evans, Lorna Finch, Lewis WS Fisher, Terry Foster, Isabel Garcia-Dorival, Philip Gunning, Catherine Hartley, Rebecca L Jensen, Christopher B Jones, Trevor R Jones, Shadia Khandaker, Katharine King, Robyn T. Kiy, Chrysa Koukorava, Annette Lake, Suzannah Lant, Diane Latawiec, Lara Lavelle-Langham, Daniella Lefteri, Lauren Lett, Lucia A Livoti, Maria Mancini, Sarah McDonald, Laurence McEvoy, John McLauchlan, Soeren Metelmann, Nahida S Miah, Joanna Middleton, Joyce Mitchell, Shona C Moore, Ellen G Murphy, Rebekah Penrice-Randal, Jack Pilgrim, Tessa Prince, Will Reynolds, P. Matthew Ridley, Debby Sales, Victoria E Shaw, Rebecca K Shears, Benjamin Small, Krishanthi S Subramaniam, Agnieska Szemiel, Aislynn Taggart, Jolanta Tanianis-Hughes, Jordan Thomas, Erwan Trochu, Libby van Tonder, Eve Wilcock, J. Eunice Zhang, Lisa Flaherty, Nicole Maziere, Emily Cass, Alejandra Doce Carracedo, Nicola Carlucci, Anthony Holmes, Hannah Massey; *Edinburgh Laboratory Staff and Volunteers*: Lee Murphy, Sarah McCafferty, Richard Clark, Angie Fawkes, Kirstie Morrice, Alan Maclean, Nicola Wrobel, Lorna Donnelly, Audrey Coutts, Katarzyna Hafezi, Louise MacGillivray, Tammy Gilchrist; *Local Principal Investigators*: Kayode Adeniji, Daniel Agranoff, Ken Agwuh, Dhiraj Ail, Erin L. Aldera, Ana Alegria, Sam Allen, Brian Angus, Abdul Ashish, Dougal Atkinson, Shahedal Bari, Gavin Barlow, Stella Barnass, Nicholas Barrett, Christopher Bassford, Sneha Basude, David Baxter, Michael Beadsworth, Jolanta Bernatoniene, John Berridge, Colin Berry, Nicola Best, Pieter Bothma, David Chadwick, Robin Brittain-Long, Naomi Bulteel, Tom Burden, Andrew Burtenshaw, Vikki Caruth, David Chadwick, Duncan Chambler, Nigel Chee, Jenny Child, Srikanth Chukkambotla, Tom Clark, Paul Collini, Catherine Cosgrove, Jason Cupitt, Maria-Teresa Cutino-Moguel, Paul Dark, Chris Dawson, Samir Dervisevic, Phil Donnison, Sam Douthwaite, Andrew Drummond, Ingrid DuRand, Ahilanadan Dushianthan, Tristan Dyer, Cariad Evans, Chi Eziefula, Chrisopher Fegan, Adam Finn, Duncan Fullerton, Sanjeev Garg, Sanjeev Garg, Atul Garg, Effrossyni Gkrania-Klotsas, Jo Godden, Arthur Goldsmith, Clive Graham, Elaine Hardy, Stuart Hartshorn, Daniel Harvey, Peter Havalda, Daniel B Hawcutt, Maria Hobrok, Luke Hodgson, Anil Hormis, Michael Jacobs, Susan Jain, Paul Jennings, Agilan Kaliappan, Vidya Kasipandian, Stephen Kegg, Michael Kelsey, Jason Kendall, Caroline Kerrison, Ian Kerslake, Oliver Koch, Gouri Koduri, George Koshy, Shondipon Laha, Steven Laird, Susan Larkin, Tamas Leiner, Patrick Lillie, James Limb, Vanessa Linnett, Jeff Little, Mark Lyttle, Michael MacMahon, Emily MacNaughton, Ravish Mankregod, Huw Masson, Elijah Matovu, Katherine McCullough, Ruth McEwen, Manjula Meda, Gary Mills, Jane Minton, Mariyam Mirfenderesky, Kavya Mohandas, Quen Mok, James Moon, Elinoor Moore, Patrick Morgan, Craig Morris, Katherine Mortimore, Samuel Moses, Mbiye Mpenge, Rohinton Mulla, Michael Murphy, Megan Nagel, Thapas Nagarajan, Mark Nelson, Lillian Norris, Matthew K. O’Shea, Igor Otahal, Marlies Ostermann, Mark Pais, Carlo Palmieri, Selva Panchatsharam, Danai Papakonstantinou, Hassan Paraiso, Brij Patel, Natalie Pattison, Justin Pepperell, Mark Peters, Mandeep Phull, Stefania Pintus, Jagtur Singh Pooni, Tim Planche, Frank Post, David Price, Rachel Prout, Nikolas Rae, Henrik Reschreiter, Tim Reynolds, Neil Richardson, Mark Roberts, Devender Roberts, Alistair Rose, Guy Rousseau, Bobby Ruge, Brendan Ryan, Taranprit Saluja, Matthias L Schmid, Aarti Shah, Prad Shanmuga, Anil Sharma, Anna Shawcross, Jeremy Sizer, Manu Shankar-Hari, Richard Smith, Catherine Snelson, Nick Spittle, Nikki Staines, Tom Stambach, Richard Stewart, Pradeep Subudhi, Tamas Szakmany, Kate Tatham, Jo Thomas, Chris Thompson, Robert Thompson, Ascanio Tridente, Darell Tupper-Carey, Mary Twagira, Nick Vallotton, Rama Vancheeswaran, Lisa Vincent-Smith, Shico Visuvanathan, Alan Vuylsteke, Sam Waddy, Rachel Wake, Andrew Walden, Ingeborg Welters, Tony Whitehouse, Paul Whittaker, Ashley Whittington, Padmasayee Papineni, Meme Wijesinghe, Martin Williams, Lawrence Wilson, Sarah Cole, Stephen Winchester, Martin Wiselka, Adam Wolverson, Daniel G Wootton, Andrew Workman, Bryan Yates, Peter Young.

## Author contributions

EP, JT, KJR, SI, FM, KK, CD, EV, SD, KJ, and NJC performed experiments. EP, JT, KJR, SI, TE, LL, EV, SCM, SD, WP and GP analyzed laboratory and clinical data. NC, JH and PC designed, produced and donated key reagents. LL, CJ, HH, EV, SEM and SCM administered patient specimens and curated clinical data. MS, JB and PO designed and delivered the ISARIC4C consortium project. The study was designed by SI, MM, JB, PO, MS, WP, RT, and GP. The manuscript was written by EP, JT, KJR, SI, TE, LT, AH, MS, WP, RT. and GP. All authors contributed to the article and approved the submitted version.
