# Supplementary material for: SARS-CoV-2 antibody responses associate with sex, age and disease severity in previously uninfected people admitted to hospital with COVID-19: An ISARIC4C prospective study
Source: Front Immunol. 2023 Mar 15;14:1146702. doi: 10.3389/fimmu.2023.1146702 (PMC10087108; doi:10.3389/fimmu.2023.1146702)
Supplement: Supplementary file 1 [file DataSheet_1.pdf]

Cohort	
Participants	337
Male	210 (63%)
Female	127 (37%)

Age	
All	57(15-94)
Male	57(20-90)
Female	58(15-94)

Severity	n	Male	Female
1	130	66(51%)	64(49%)
2	73	44(60%)	29(40%)
3	34	23(68%)	11(31%)
4	59	41(69%)	18(31%)
5	37	34(92%)	3(8%)

Severity Score definition				
Oxygen?	NIV or HFNO?	Invasive Ventilation?	Death < 28 days?	Score
NO	NO	NO	NO	1
YES	NO	NO	NO	2
YES	YES	NO	NO	3
NO	YES	NO	NO	3
YES	YES	YES	NO	4
YES	NO	YES	NO	4
YES	YES	YES	YES	5
YES	YES	NO	YES	5
YES	NO	YES	YES	5
YES	NO	NO	YES	5

Supplementary Figure 1 Cohort population, demographics and disease severity groupings.

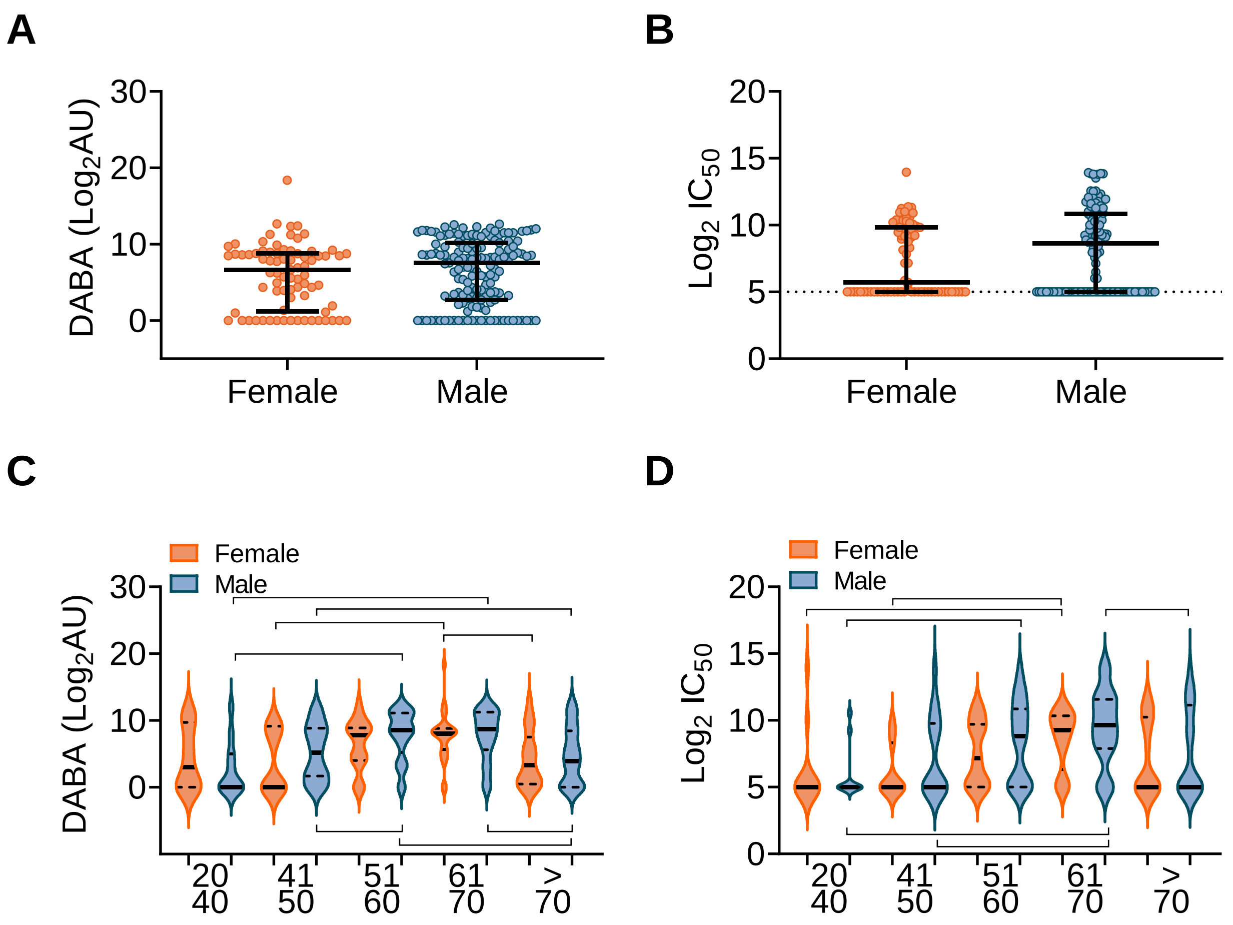

Supplementary Figure 2 Ab responses from patient samples at time of recruitment split into age groupings and sex. (**A**) Dot plot comparing anti-RBD binding responses in females (orange) vs males (blue). (**B**) Dot plot comparing neutralising antibody levels in females (orange) and males (blue). (**C**) Total anti-RBD binding responses split into age groupings and females (orange) versus males (blue). (**D**) Ab neutralisation (IC_{50}) binding responses split into age groupings and female (green) versus male (blue). Statistically significant differences (non-parametric ANOVA (Kruskal-Wallis test) indicated by horizontal lines above or below the groupings.

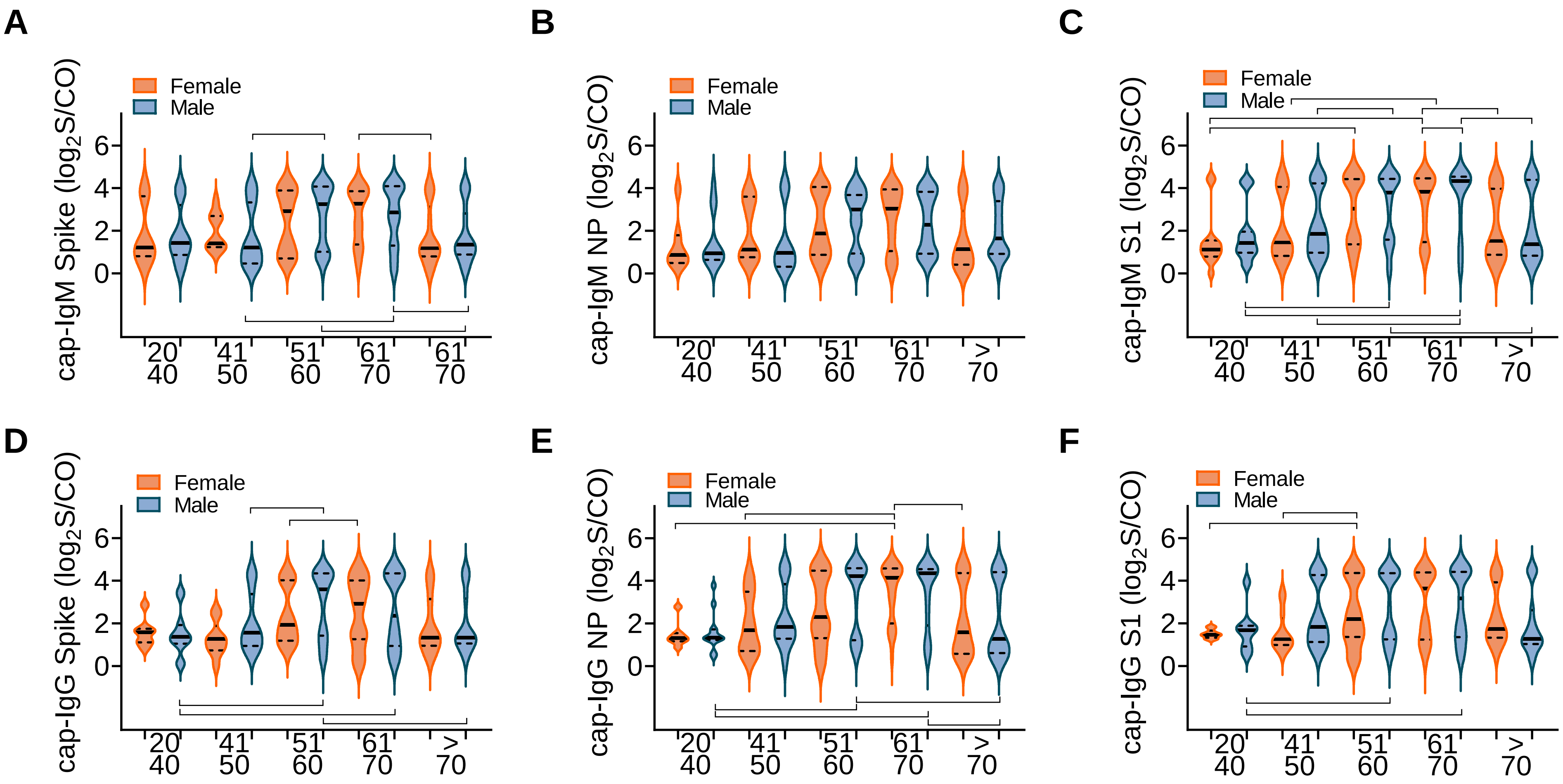

Supplementary Figure 3 Ab subclass binding responses, measured from samples taken at patient recruitment, against Spike, NP and S1 antigens in relation to age and sex. IgM antibody binding responses against spike **(A)**, NP **(B)** and S1 **(C)** antigen (orange female and blue male). IgG antibody binding responses against Spike **(D)**, NP **(E)** and S1 **(F)** antigen (female in orange and male in blue). In all panels mean values and confidence intervals shown (black lines) and statistically significant differences (non-parametric ANOVA (Kruskal-Wallis test) indicated by horizontal lines above or below the groupings.

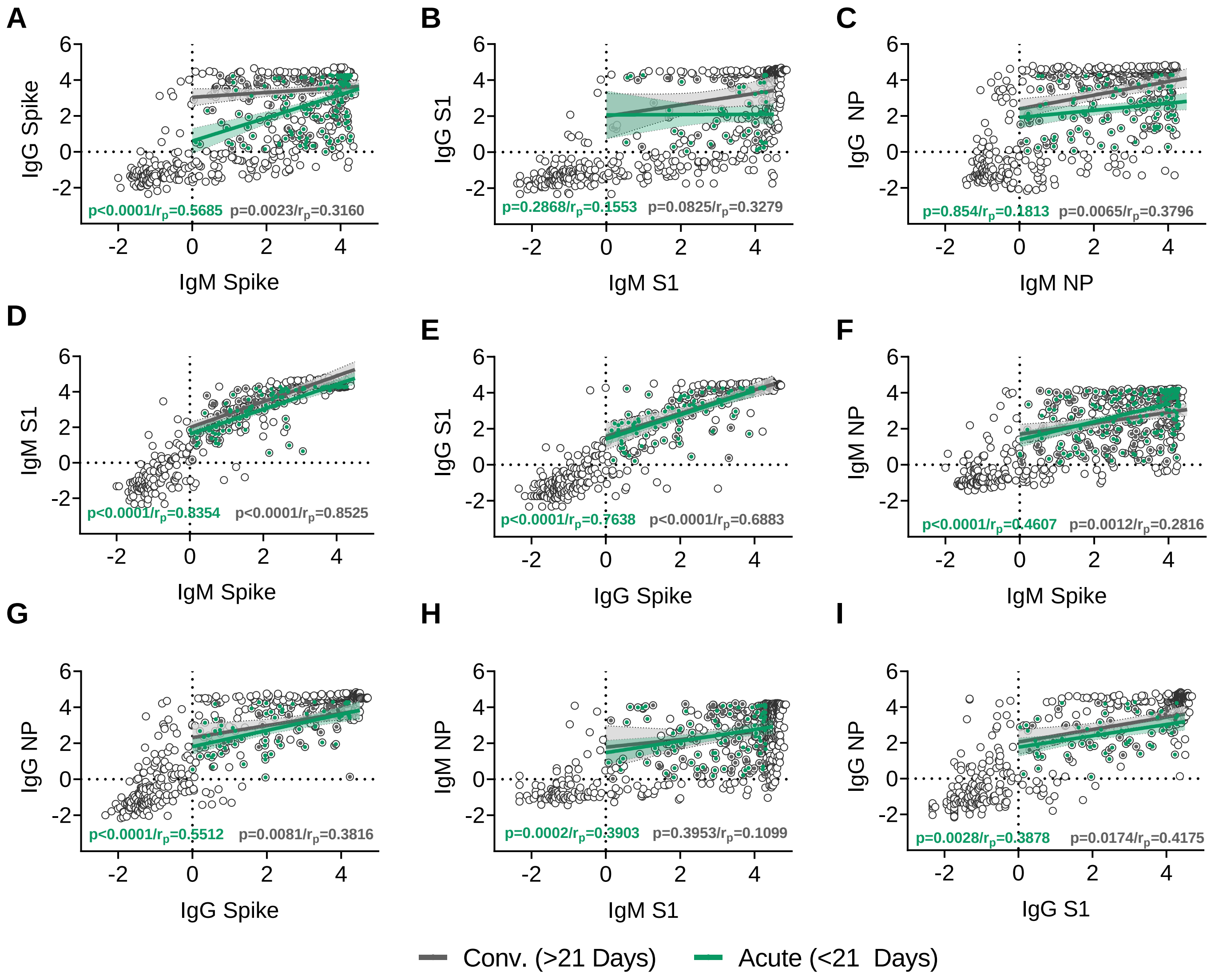

Supplementary Figure 4 Association of antibody binding responses between variant antigens and antibody subclasses. Binding plots describing correlation between antibody measurements taken during acute infection (less than 21 days from symptom onset, green) and convalescence (more than 21 days from symptom onset, grey). (A) Correlation between IgG and IgM antibody responses directed against spike. **(B)** Correlation between IgG and IgM antibody responses directed against S1. **(C)** Correlation between IgG and IgM antibody responses directed against NP. **(D)** Correlation between IgM antibody responses directed against S1 and spike. **(E)** Correlation between IgG antibody responses directed against S1 and spike. **(F)** Correlation between IgM antibody responses directed against NP and spike. **(G)** Correlation between IgG antibody responses directed against NP and spike. **(H)** Correlation between IgM antibody responses directed against NP and S1. **(G)** Correlation between IgG antibody responses directed against NP and S1. The black doted lines depict the detection limit for antibody type responses. The spearman correlation is represented by green (acute) and black (convalescence) lines. The p and rp values are provided in each panel in corresponding colours.

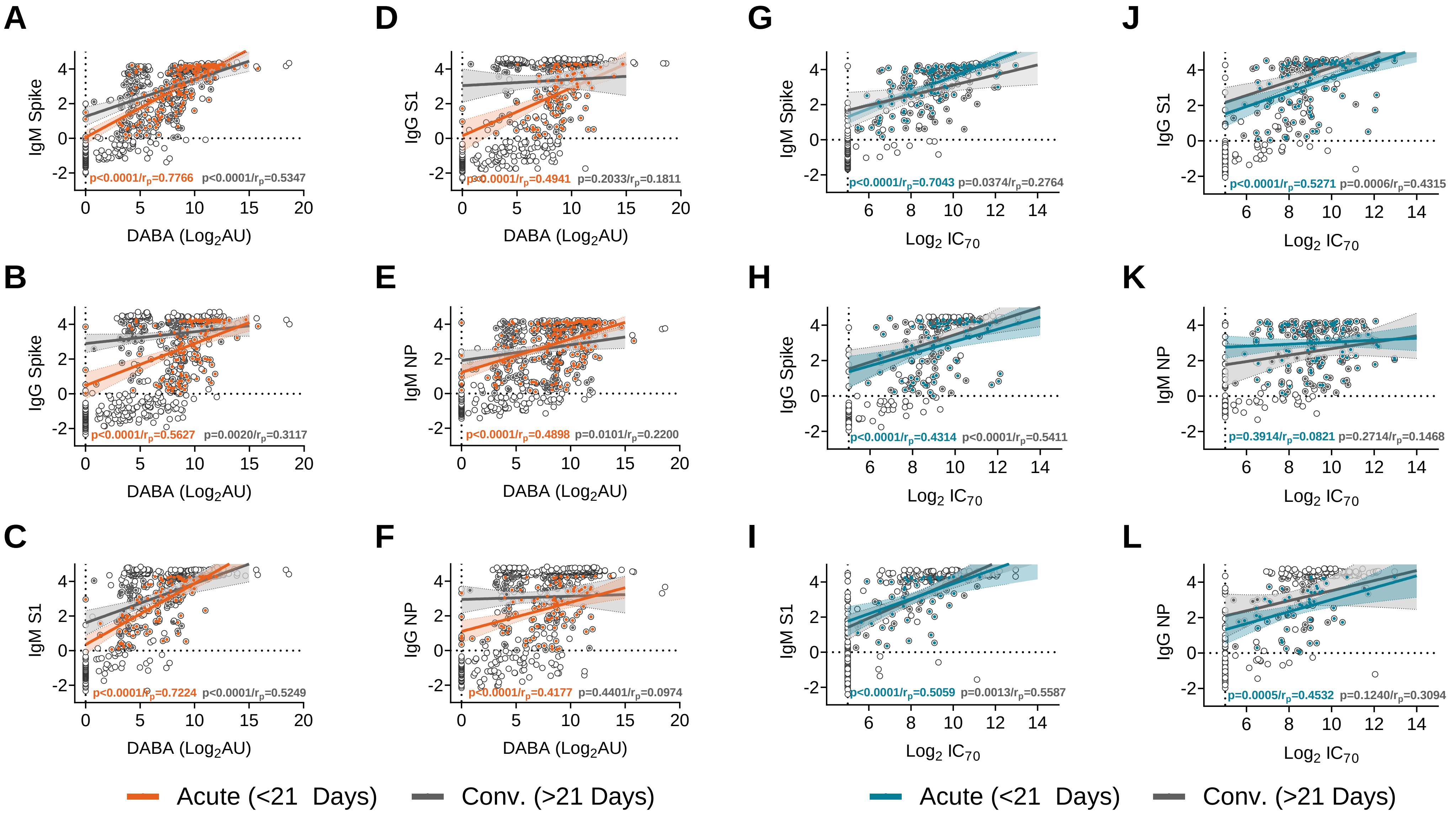

Supplementary Figure 5 Association of binding Ab titres with total (DABA) or neutralising Ab responses. (A-F) Association of anti-RBD titres with antibody responses to spike **(A and B)**, S1 **(C and D)** and NP **(E and F)**. The spearman correlation (acute phase/ less than 21 days from symptom onset) is shown in orange and during convalescence shown in grey. (G-L). Association of neutralising antibody (IC_{70}) titres with binding antibody responses to spike **(G and H)**, SI **(I and J)** and NP **(K and L)**. The spearman correlation (acute phase/ less than 21 days from symptom onset) is shown in blue for and during convalescence shown in grey. The black doted lines depict the detection limit for antibody type responses. The p and rp values are provided in each panel in corresponding colours.

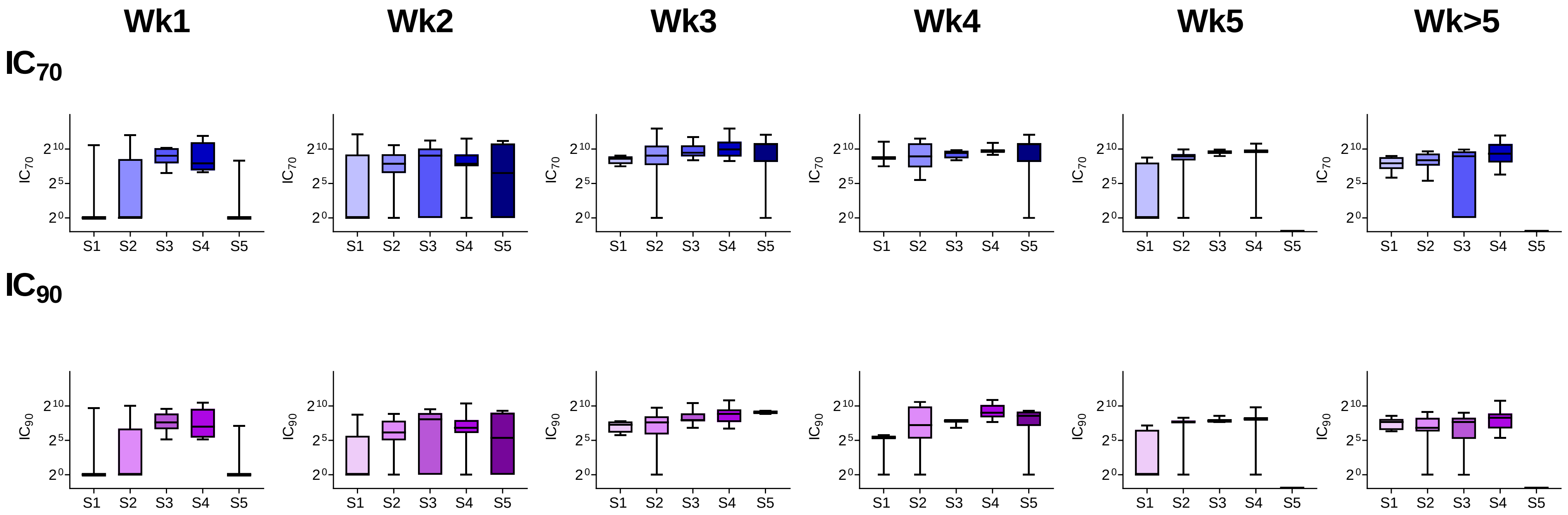

Supplementary Figure 6 Neutralizing Ab responses split into disease severity groupings and weeks following enrolment. (**A**) IC_{70} and (**B**) IC_{90} neutralisation responses split into disease severity and week of infection (wk1->wk5). Statistically significant differences (non-parametric ANOVA (Kruskal-Wallis test) indicated by horizontal lines above the groupings.

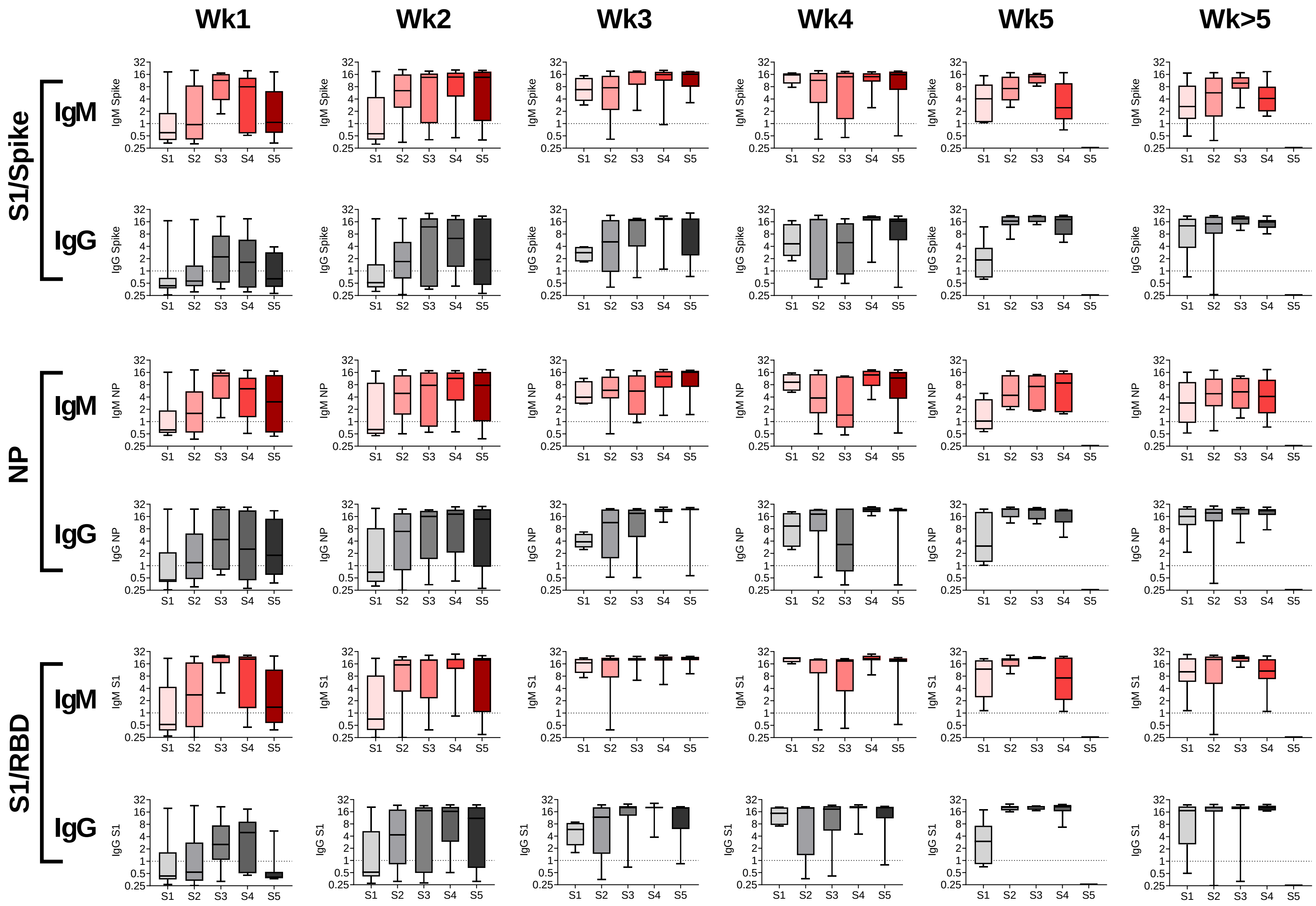

Supplementary Figure 7 IgM and IgG Ab binding responses raised against spike, NP and S1 antigen. The antibody titres were grouped according to severity (S1-S5) and were compared at every week following disease onset (wk1->wk5). Statistically significant differences (non-parametric ANOVA (Kruskal-Wallis test) are indicated by horizontal lines above the groupings.

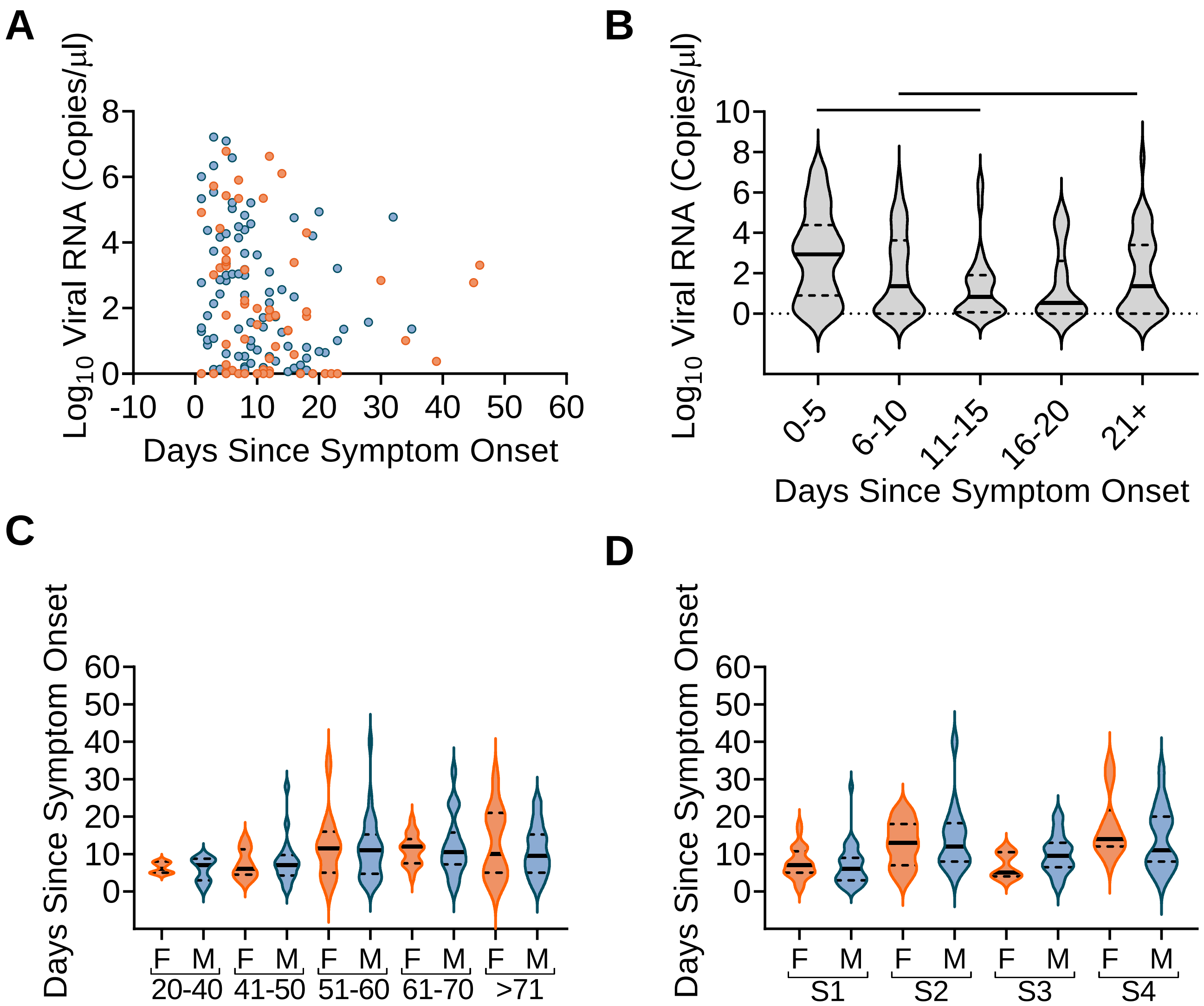

Supplementary Figure 8 Upper respiratory tract viral load by days since disease onset, sex, age and severity group. (**A**) Viral load at recruitment plotted against days since disease onset split into female (orange) and male (blue). (**B**) Viral load grouped in time since disease onset. (**C**) Days since disease onset, for the subgroup where viral load was available, split into age groupings and female (orange) versus male (blue). (**D**) Days since disease onset grouped according to disease severity and female (orange) and male (blue) shown.
